# Long-Read Sequencing for the Rapid Response to Infectious Diseases Outbreaks

**DOI:** 10.1007/s40588-025-00247-y

**Published:** 2025-05-15

**Authors:** Josephine B. Oehler, Kaitlin Burns, Jeffrey Warner, Ulf Schmitz

**Affiliations:** 1 Computational Biomedicine Lab, College of Science and Engineering, James Cook University, 1 James Cook Drive, Townsville, QLD 4811 Australia; 2 College of Medicine and Dentistry, James Cook University, Townsville, QLD Australia; 3 Australian Institute of Tropical Health and Medicine, James Cook University, Cairns, Australia; 4 Centre for Tropical Bioinformatics and Molecular Biology, James Cook University, Cairns, Australia; 5 Centenary Institute, The University of Sydney, Camperdown, Australia

**Keywords:** Third-generation sequencing, Pathogen identification, Genomic epidemiology antimicrobial resistance profiling, Metagenomics, Real-time surveillance

## Abstract

**Purpose of Review:**

Long-read sequencing (LRS) has revolutionized pathogen surveillance by enabling real-time, high-fidelity genomic analysis critical for outbreak response. This review synthesizes recent breakthroughs in LRS, evaluating its impact on genomic epidemiology, metagenomics, and public health decision-making while addressing limitations and prospects for integrating LRS into global outbreak surveillance.

**Recent Findings:**

Unlike short-read sequencing, LRS—pioneered by Oxford Nanopore Technologies (ONT) and Pacific Biosciences (PacBio)—resolves complex genomic structures, antimicrobial resistance determinants, and transmission dynamics with unprecedented accuracy. The portability of some LRS devices has facilitated rapid pathogen identification in field settings, notably during the Ebola and COVID-19 pandemics. Despite advancements in basecalling algorithms and target enrichment, challenges including sequencing errors, computational bottlenecks, and cost barriers remain.

**Summary:**

By critically evaluating recent findings and discussing future directions, this review highlights the importance of leveraging LRS for outbreak preparedness and response, equipping researchers and public health professionals with the knowledge necessary to navigate the complexities of modern infectious disease challenges.

## Introduction

The ability to characterize pathogens rapidly and accurately during an outbreak is critical for effective public health response. Advances in genomic sequencing have revolutionized infectious disease surveillance, with long-read sequencing (LRS) emerging as a transformative technology. Unlike short-read sequencing, which relies on fragmented DNA assembly, LRS — pioneered by platforms including Oxford Nanopore Technologies (ONT) and Pacific Biosciences (PacBio) — enables the generation of contiguous, high-fidelity genomic sequences with unprecedented resolution [1, 2]. This capability is particularly valuable for outbreak investigations, where rapid identification of virulence factors, antimicrobial resistance genes, and transmission dynamics is essential for guiding public health interventions.

Recent developments in LRS have accelerated our ability to decode complex microbial genomes, resolve structural variations, and analyse epigenetic modifications in real time [3]. These advances are reshaping our understanding of pathogen evolution, host–pathogen interactions, and the mechanisms underlying outbreak propagation [4]. Notably, the portability of nanopore sequencing instruments has allowed real-time genomic surveillance in field settings, offering a powerful tool for outbreak response in resource-limited environments [5]. Despite these remarkable achievements, challenges remain, including sequencing error rates, data analysis bottlenecks, and cost considerations that may limit widespread adoption.

This review aims to synthesize the latest research on LRS for outbreak response, highlighting key breakthroughs, methodological innovations, and ongoing debates in the field. In particular, we will examine the role of LRS in enhancing genomic epidemiology, its applications in emerging infectious diseases, and the integration of real-time sequencing data with public health decision-making. Additionally, we will address controversial topics, such as the accuracy of long-read technologies compared to short-read approaches, the feasibility of their large-scale deployment, and ethical considerations surrounding pathogen genome sequencing during public health crises [6].

## Technological Background and New Developments

LRS has advanced pathogen genomics by enabling the characterization of entire genomes, including regions that were previously inaccessible with short-read technologies [4]. Unlike second-generation sequencing (dominated by Illumina), which produces reads of short DNA fragments (typically 50–300 base pairs), LRS platforms can generate reads spanning tens to hundreds of kilobases [7]. These ultra-long reads facilitate complete genome assemblies, detection of large structural variants, and characterization of epigenetic modifications—all critical factors in understanding the genetic dynamics of infectious agents during outbreaks [8].

One of the most impactful applications of LRS is its ability to resolve highly repetitive and structurally complex regions of microbial genomes. For example, in *Mycobacterium tuberculosis*, LRS has been instrumental in accurately identifying large insertions and deletions (indels), which play a crucial role in drug resistance evolution [9]. Similarly, *Plasmodium falciparum*, the malaria-causing parasite, has a genome characterized by extensive segmental duplications and subtelomeric gene families involved in immune evasion [10, 11]. LRS provides a complete representation of these genomic features, overcoming limitations imposed by short-read sequencing.

Another critical advantage of LRS is its capacity to analyse epigenetic modifications directly. PacBio’s Single-Molecule Real-Time (SMRT) sequencing enables the detection of base modifications such as N6-methyladenine (6 mA) and 5-methylcytosine (5 mC), which influence bacterial virulence and host interactions [12, 13]. ONT nanopore sequencing, on the other hand, can directly sequence RNA molecules without requiring cDNA conversion, allowing for real-time (epi-)transcriptome analysis during an outbreak [14]. This capability is particularly valuable for studying RNA viruses, such as coronaviruses and influenza, in their native state.

Significant technological advancements have addressed the historical challenges of LRS, including accuracy and scalability. PacBio’s HiFi sequencing, which employs circular consensus sequencing (CCS), has reduced error rates to below 1%, making it competitive with Illumina while maintaining the benefits of LRS [15, 16]. ONT’s latest chemistry, coupled with deep learning-based basecalling algorithms like *Dorado* (github.com/nanoporetech/dorado) and *Bonito* (github.com/nanoporetech/bonito), has dramatically improved base accuracy, achieving Q20 + (99% accuracy) in nanopore reads [17–19]. Furthermore, ONT’s target enrichment methods including adaptive sampling [20], CRISPR-Cas9-guided amplification-free enrichment[21], 16S barcoding [22], and amplicon sequencing [23] allow selective enrichment of specific genomic regions in real-time, reducing sequencing costs and improving efficiency in metagenomic studies [15].

Bioinformatics innovations have further enhanced the utility of LRS. Hybrid assembly pipelines, such as *Unicycler* [24] and *Flye* [25] integrate short- and long-read data to produce high-quality genome assemblies with improved contiguity and accuracy. Deep-learning frameworks, including *Medaka* (github.com/nanoporetech/medaka) for ONT and *DeepConsensus* [21] for PacBio, enhance raw read correction, reducing systematic errors [26]. Cloud-based analysis tools, such as *Nextstrain* [27]* a*nd *EDGE Bioinformatics* [28] now allow real-time epidemiological modelling, enabling rapid genomic-informed public health decision-making during outbreaks [29].

LRS is well positioned to become a cornerstone of infectious disease surveillance and outbreak response. As the technology matures, its ability to provide rapid, high-resolution genomic data will revolutionize our ability to detect, track, and mitigate emerging pathogen threats in real time (Table 1).

**Table 1:** Key advantages, limitations, and technological advancements of long-read sequencing for outbreak response

Aspect	Advantages	Limitations	Recent advancements
Pathogen identification	Rapid, real-time sequencing in field settings (e.g., Ebola, COVID-19)[30, 31]	Sequencing errors may impact variant identification	AI-driven basecalling improving accuracy
Genomic epidemiology	Resolves complex transmission dynamics within outbreaks [31]	Requires bioinformatics expertise for interpretation	Hybrid assembly pipelines enhance accuracy
Antimicrobial resistance profiling	Full-length plasmid and resistance gene identification [32]	Cost-intensive for routine hospital use	CRISPR-Cas9-based target enrichment for AMR genes [32]
Metagenomics	Direct sequencing of mixed microbial communities [33]	Computationally demanding; reference database biases	Machine learning-based taxonomic classification tools
Real-time surveillance	Portable sequencers enable on-site outbreak monitoring [34]	Internet access needed for cloud-based analysis	Mobile-compatible sequencing tools (MinION Mk1D)[34]
Epigenetics & transcriptomics	Detects methylation patterns and RNA viruses directly [35]	Higher error rates in modified base detection	PacBio HiFi & ONT direct RNA sequencing improving resolution [35]
Scalability & accessibility	Field-deployable sequencing in low-resource settings	High equipment and reagent costs in developing regions	Development of cost-effective, miniaturized sequencing devices
Data integration & public health impact	Enables rapid policy decisions and outbreak tracking [31]	Lack of standardized protocols for global data sharing [36]	for real-time epidemiology

## Applications of LRS in Infectious Diseases Outbreaks

**Fig. 1:**
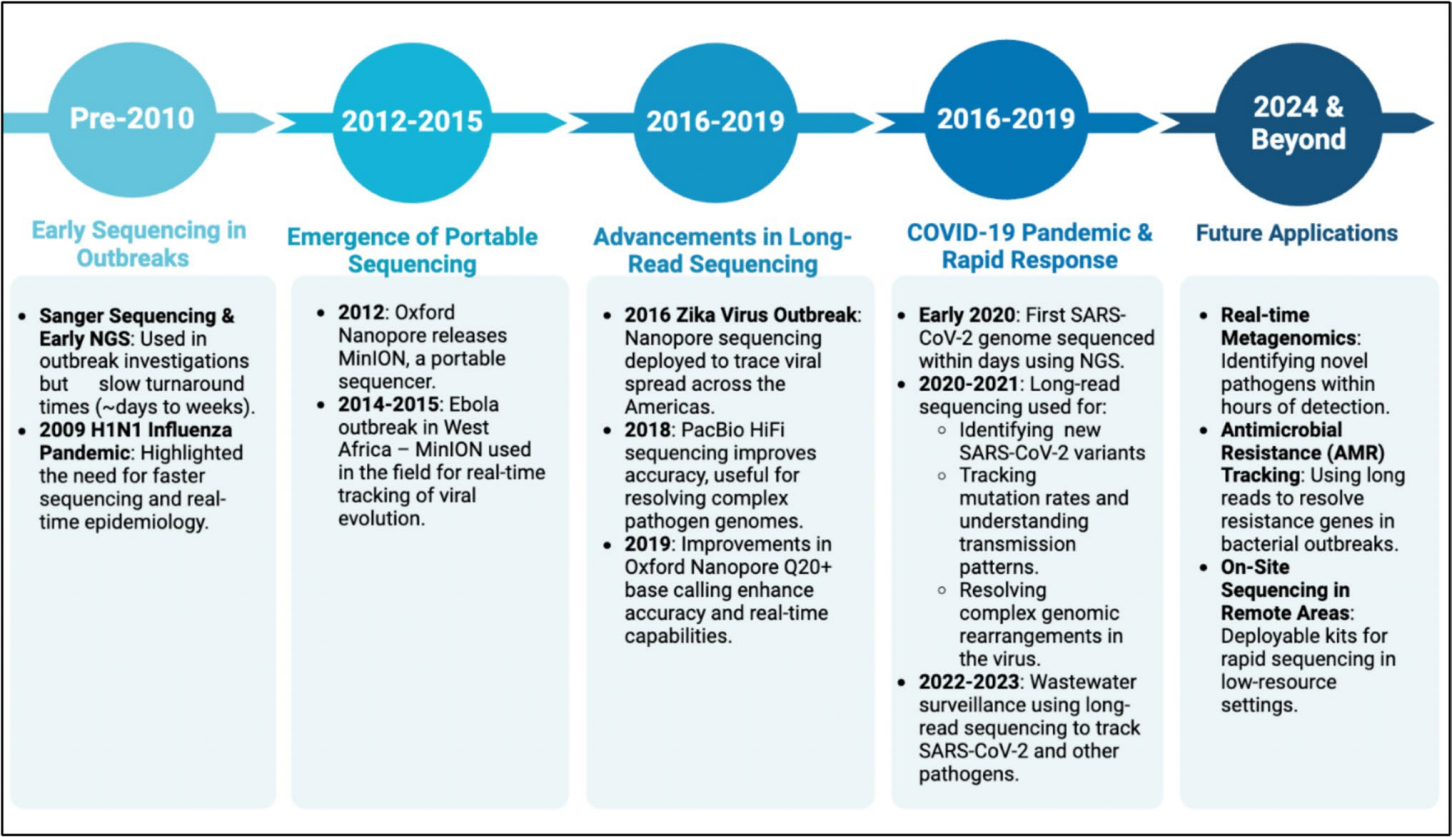
Evolution of sequencing technologies in outbreak response (Pre-2010 to Present)

Applications of LRS have emerged as powerful tools in infectious diseases outbreaks, ranging from pathogen identification, genomic epidemiology, antimicrobial resistance (AMR) profiling, to real-time monitoring of viral evolution (Fig. 1).

### Pathogen Identification and Genomic Epidemiology

LRS has significantly enhanced the ability to identify pathogens during outbreaks. For instance, during the 2014–2016 Ebola outbreak in West Africa, the use of the MinION sequencer from ONT enabled rapid sequencing of the Ebola virus, allowing researchers to trace transmission routes and identify viral variants within hours of sample collection [37, 38]. This capability was crucial for informing public health interventions and controlling the spread of the virus. Similarly, in the context of the 2020 COVID-19 outbreak, LRS has played a pivotal role in tracking the evolution of SARS-CoV [39, 40]. Studies utilizing ONT sequencing have provided insights into the emergence of variants of concern, such as the Delta and Omicron variants, by analysing mutations associated with increased transmissibility and immune escape [41–43]. The ability to generate high-quality genomic data in real-time has allowed public health authorities to respond swiftly to emerging threats, adjusting vaccination strategies and public health measures accordingly.

Nanopore LRS is particularly valuable in resource-limited regions for genomic surveillance [44]. The high acquisition cost, complex workflows, computational demands, and required bioinformatics expertise pose significant challenges for small and medium-sized laboratories adopting next-generation sequencing, especially in remote areas and low- and middle-income countries (LMICs) [44, 45]. ONT addresses these barriers with its affordability, portability, and cost efficiency [18, 44]. A key pathogen disproportionately affecting these regions is *Burkholderia pseudomallei*, an environmental, Gram-negative bacterium endemic to tropical and subtropical regions [46, 47]. This organism is the causative agent of melioidosis, a potentially fatal disease [48]. Implementing cost-efficient, accurate genotyping methods in these areas could enhance pathogen surveillance and outbreak response, as demonstrated during the SARS-CoV pandemic [39–43]. A core genome multilocus sequence typing (cgMLST) method has been developed to investigate both environmental and clinical *B. pseudomallei* isolates, demonstrating its effectiveness in tracking environmental reservoirs of infection [49]. Optimisation of nanopore sequencing for accurate cgMLST of *B. pseudomallei* has been achieved, with improvements such as native barcoding, and using the latest models for superaccurate basecalling (e.g. sup@v5.0 in *Dorado*) and polishing (e.g. medaka_g360_HAC in *Medaka*), reducing discrepancies between nanopore LRS and short-read sequencing to a maximum of two allele variations per isolate across the datasets [50]. Both approaches have focused on simplifying workflows, improving cost efficiency, and enhancing typing accuracy to facilitate implementation in LMICs, ultimately strengthening genomic surveillance capacity [49, 50].

### Antimicrobial Resistance Profiling

The rise of antimicrobial resistance poses a significant challenge to public health, and LRS has proven invaluable in profiling AMR genes in pathogens. Traditional short-read sequencing often struggles to accurately resolve complex genomic regions, particularly those harbouring resistance genes on plasmids [51]. In contrast, LRS can provide complete assemblies of plasmid genomes, facilitating the identification of resistance determinants and their genetic contexts [52]. For example, a study by Roberts et al. demonstrated that ONT LRS enabled the generation of complete bacterial and plasmid genomes without the need for short-read sequencing, allowing for accurate localization of antimicrobial resistance genes [36]. This capability is particularly important in understanding the mechanisms of resistance transmission, as plasmids can facilitate horizontal gene transfer among bacterial populations [53]. Moreover, the Bac-PULCE method, which employs CRISPR enrichment combined with LRS, has been developed to enhance the resolution of AMR profiling. This approach allows for the sequencing of linked AMR loci, providing insights into the genetic basis of resistance even when not all resistance genes are targeted for enrichment. Such advancements in AMR profiling are critical for informing treatment strategies and guiding public health interventions during outbreaks.

LRS-based AMR profiling of infectious agents, can also significantly reduce turnaround times and enhance drug resistance detection within a clinical setting, improving patient outcomes through rapid and tailored antibiotic intervention [54–56]. The implementation of LRS for AMR detection will enhance research on *Mycobacterium tuberculosis*. Traditional culture-based methods for *M. tuberculosis* are inherently slow, often requiring several weeks to generate results, limiting the timely detection of resistance profiles [54, 56]. Implementation of the GeneXpert MTB/RIF assay, which concurrently detects *M. tuberculosis* complex (MTBC) and resistance to rifampin (RIF), significantly reduces turnaround time to two hours for a tuberculosis diagnosis but is limited to detecting high-prevalence mutations within the 81-bp rifampin resistance-determining region (*rpoB*), reducing its ability to determine drug resistance across a broad range of antibiotics [56, 57]. Nanopore sequencing enables comprehensive resistance profiling across a wide range of anti-tuberculosis drugs, enhancing the detection of multidrug-resistant *M. tuberculosis* and supporting targeted treatment strategies [54, 55]. This approach has been successfully implemented across multiple clinical settings, where multiplex PCR was used to amplify key drug resistance-associated regions for sequencing, allowing for the rapid detection of resistance to rifampicin, isoniazid, ethambutol, streptomycin, and fluoroquinolones within twelve hours [54]. Similarly, a multicentre study in South Africa and Zambia successfully integrated a similar multiplex PCR-based nanopore LRS method into two reference laboratories for use in tuberculosis diagnostics. Before widespread implementation of this method in high tuberculosis-burden, resource-limited regions, it was recommended to address several technical and operational challenges, including reagent supply and storage, improved access to IT support, continuous training and capacity building, better working facilities, and automation—where feasible—to enhance reproducibility [55].

### Real-Time Monitoring of Viral Evolution

The ability to monitor viral evolution in real-time is another significant application of LRS in outbreak response. The rapid sequencing capabilities of ONT have allowed researchers to track the emergence of new viral variants during outbreaks. For instance, during the COVID-19 pandemic, researchers utilized LRS to monitor the genetic diversity of SARS-CoV-2, identifying mutations that may impact transmissibility and vaccine effectiveness [43]. This real-time data has been instrumental in guiding public health responses and vaccine development. Additionally, LRS has been applied to monitor other viral pathogens, such as the Zika virus and the influenza virus [58, 59]. In a study examining the Zika virus outbreak, LRS provided insights into the genetic diversity of the virus, aiding in the understanding of its transmission dynamics [60]. Such applications underscore the importance of LRS in enhancing our understanding of viral evolution and informing outbreak management strategies. However, real-time sequencing still encounters logistical barriers. Field-deployable sequencers, such as the MinION, are highly portable but still require certain lab equipment, sufficient computational resources, and stable internet access for real-time data processing. While the widely adopted MinION model Mk1 C with integrated compute is not available anymore, it’s successor, the Mk1D, can be operated with state-of-the-art mobile computers. Cloud-based platforms like *Nextstrain* have facilitated rapid genomic analysis, but data privacy concerns must be addressed before large-scale implementation in outbreak settings [61].

### Metagenomic Applications

LRS has also advanced metagenomic studies, enabling the characterization of complex microbial communities in outbreak scenarios. The ability to generate long reads facilitates the identification of diverse microbial species and their associated functions, providing valuable insights into the dynamics of infectious disease outbreaks. For example, a study by Foster-Nyarko et al. utilized LRS to investigate the genomic diversity of *Escherichia coli* isolates from non-human primates in The Gambia [62]. The researchers documented the contribution of plasmids to the genomic diversity observed in the study population, highlighting the role of horizontal gene transfer in the spread of antimicrobial resistance [62, 63]. Such metagenomic applications are crucial for understanding the ecological dynamics of pathogens during outbreaks and informing public health interventions. Despite these breakthroughs, computational limitations hinder LRS-based metagenomics. Current assembly tools struggle with complex microbiomes, and reference databases for pathogen identification remain biased toward well-characterized organisms. Developing machine-learning-driven taxonomic classifiers could enhance the accuracy of metagenomic surveillance in outbreak scenarios.

## Limitations of Long-Read Sequencing

Despite the numerous advantages of LRS, several limitations hinder its widespread adoption and feasibility in outbreak response. Addressing these challenges is essential for maximizing the potential of LRS technologies.

### Error Rates and Data Quality

One of the primary limitations of LRS is the relatively high error rates associated with the generated data. While recent advancements, such as PacBio's HiFi sequencing, have reduced error rates to below 1%, ONT sequencing still exhibits significantly higher error rates compared to short-read technologies [64]. This can lead to inaccuracies in variant calling and hinder the reliable identification of pathogen strains during outbreaks. For example, Bull et al. highlighted the importance of accurate sequence determination for defining the phylogenetic structure of SARS-CoV-2 outbreaks, noting that higher error rates in ONT sequencing could complicate this process [65, 66].

Biased sequencing errors associated with ONT LRS present an additional challenge for attaining high accuracy and reliable sequencing. ONT has difficulty in producing accurate sequencing results within regions of DNA that contain homopolymers, short tandem repeats, and DNA methylation [50, 65, 67]. Methylation-related errors were shown to produce non-reproducible typing results between laboratories investigating four public-health relevant bacteria (*Enterococcus faecium, Klebsiella pneumoniae, Listeria monocytogenes*, and *Staphylococcus aureus*) as part of a multicentre study assessing the implementation of nanopore sequencing for genomic surveillance [67]. Further investigation identified consistent DNA motifs associated with these methylation-related errors [67]. Careful analysis is required when whole genome sequencing (WGS) novel or problematic bacterial strains due to these biased sequencing errors, complicating the implementation of nanopore LRS for widespread genomic surveillance [50, 65, 67].

### Computational Challenges

The analysis of LRS data presents significant computational challenges. The bioinformatics tools required to process and interpret long-read data are still evolving [68], and many existing tools are optimized for short-read data [69]. Hybrid assembly approaches, which combine short- and long-read data, can complicate the analysis pipeline and require additional computational resources [18, 70]. Furthermore, the integration of long-read data into existing epidemiological frameworks remains a challenge, as many public health systems are not yet equipped to handle the complexities of this data type.

### Standardization and Protocol Development

The lack of standardized protocols for LRS and LRS data analysis can lead to variability in results across different laboratories and studies. This inconsistency can hinder the comparability of data and complicate collaborative efforts in outbreak investigations. For instance, the study by Trigodet et al. emphasized the need for careful DNA extraction and library preparation to ensure the isolation of the longest molecules possible for LRS [71]. Variability in bioinformatics tools used can significantly impact sequence quality and bacterial strain identification as demonstrated by Weigl et al. [50]. The lack of standardized protocols limits the broader application of LRS in public health.

### Cost and Accessibility

Although the costs of LRS technologies have decreased, they can still be prohibitive for some laboratories, particularly in low-resource settings. The initial investment in sequencing equipment, along with the ongoing costs of reagents and consumables, can limit the widespread adoption of these technologies in outbreak response scenarios [72]. Moreover, the need for trained personnel to operate sequencing equipment and analyse data further complicates access in resource-limited environments. High-molecular-weight (HMW) DNA protocols can be technically demanding and inconsistent, and the quality of input material can significantly affect sequencing outcomes. This variability introduces complexity in generating high-quality, reproducible data. The requirement for skilled personnel to operate the instruments and interpret the data adds another layer of difficulty in resource-limited environments, further constraining the equitable implementation of LRS in global public health efforts.

### Real-Time Application Limitations

While LRS offers the potential for real-time data generation, the turnaround time for data analysis can still be a bottleneck. For example, while ONT sequencing can produce results within hours, the subsequent data processing and interpretation may take additional time, which can be critical during fast-moving outbreaks [73]. This delay can hinder timely public health interventions.

### Infrastructure and Technical Expertise

Many developing countries lack the necessary laboratory infrastructure and bioinformatics expertise required for LRS implementation [74]. Power outages, unreliable internet access, and the absence of high-performance computing facilities further limit the feasibility of real-time genomic surveillance [26]. Additionally, the training required to operate and analyse LRS data remains a critical barrier.

Moreover, the access to sequencing reagents and consumables is often restricted in developing regions due to complex supply chain challenges. Reagents often require specialized storage conditions, such as ultra-low temperatures, which can be difficult to maintain in tropical climates or conflict-affected areas [73].

## Future Prospects and Recommendations

The rapid evolution of LRS technologies has paved the way for transformative applications in outbreak response, yet several challenges remain that must be addressed for widespread global adoption. As we look toward the future, integrating LRS into routine public health surveillance will require advancements in sequencing accuracy, cost reduction, infrastructure development, and ethical considerations. This section explores cutting-edge developments and provides strategic recommendations for optimizing LRS in outbreak preparedness and response on a global scale.

### Enhancing Accuracy and Error Correction

Despite significant improvements in sequencing fidelity, LRS platforms still face challenges related to sequencing errors, particularly in low complexity regions (homopolymeric and repetitive regions) and native DNA methylation. Future developments must focus on:

- **Refining Basecalling Algorithms:** Deep learning-based tools such as *Dorado*, *Bonito* (ONT) [19], and *DeepConsensus* (PacBio) (21) have significantly improved error correction, yet further optimization using generative AI models such as RUBICON can enhance base accuracy beyond Q30 [75].
- **Refining Polishing Strategies:** Enhanced basecalling algorithms reduce error rates in bacterial nanopore sequencing but remain too high for reliable strain typing [50]. Polishing models, e.g. used by *Medaka*, can further minimise cgMLST discrepancies between LRS and short-read sequencing, as demonstrated by Weigl et al. with the use of the medaka_g360_HAC model [50].
- **PCR Preamplification:** Native DNA methylation hinders accurate and reproducible bacterial typing [50, 67]. PCR preamplification can be used to effectively remove methylation from the DNA before sequencing and resolve the methylation-related typing problems. However, PCR preamplification is only realistically suitable for sequencing of specific target regions within a bacterial genome rather than WGS [67].
- **Hybrid Sequencing Approaches:** Combining short-read sequencing (SRS) with LRS can mitigate error rates while retaining the long-range genomic context. Advances in hybrid assembly pipelines such as *Flye* [25], *Canu* [76], and *Tricycler* [77] should be leveraged for high-confidence pathogen genome reconstruction [78, 79].
- **Third-Generation Polymerases:** The development of engineered polymerases with enhanced fidelity for nanopore sequencing could reduce systematic errors, improving the reliability of pathogen identification [80].

### Reducing Costs and Expanding Accessibility

One of the primary barriers to widespread adoption of LRS, particularly in resource-limited settings, is the cost of sequencing equipment, reagents, and data analysis. Strategies for cost reduction include:

- **Portable and Affordable Sequencing Devices:** Miniaturized sequencers, such as ONT’s MinION and the low-cost flowcell Flongle, offer cost-effective alternatives to larger platforms. Further investment in developing ultra-low-cost portable sequencers could democratize access to genomic surveillance [26]. ONT is currently developing TraxION™, a compact all-in-one device designed to streamline the entire sequencing process, from nucleic acid extraction and library preparation to nanopore sequencing. This portable system offers an alternative to traditional laboratory equipment. However, ONT’s previous attempt at automating DNA extraction and library preparation in a handheld device, VolTRAX, has since been discontinued.
- **Open-Source Bioinformatics Tools:** Expanding access to free, cloud-based bioinformatics platforms (e.g., *Galaxy*, *Nextstrain* [27]*, EDGE Bioinformatics* [28]*, and Center for Genomic Epidemiology*) can eliminate the need for expensive local computing infrastructure [81].
- **Decentralized Manufacturing of Reagents:** Establishing regional production hubs for sequencing consumables can reduce dependency on global supply chains, ensuring sustainability in low-income countries [82].

### Integrating LRS into Global Genomic Surveillance Networks

For LRS to be effectively deployed in outbreak response, it must be integrated into existing global infectious disease surveillance frameworks. This requires:

- **Standardized Protocols:** Developing universally accepted guidelines for LRS-based pathogen genome sequencing, including sample preparation, sequencing workflows, and data analysis [83].
- **Interoperable Data-Sharing Platforms:** Strengthening collaborations between institutions such as the World Health Organisation (WHO), Centers for Disease Control and Prevention (CDC), and the Global Initiative on Sharing All Influenza Data (GISAID) to ensure seamless data exchange and rapid outbreak detection. Blockchain-based data-sharing models could be explored to enhance transparency and security [82, 84].
- **Real-Time Mobile Sequencing Labs:** Deploying portable sequencing units equipped with LRS technology in high-risk regions can enable on-site genomic surveillance, reducing diagnostic turnaround times during outbreaks [83].

### AI-Driven Bioinformatics and Automation

The increasing volume of genomic data generated by LRS necessitates advanced computational solutions to facilitate rapid analysis. Future directions should focus on:

- **Automated Pathogen Detection Pipelines:** AI-driven frameworks such as *MetaMaps* [13] and *Kraken2* [13] can accelerate real-time identification of outbreak pathogens from complex metagenomic samples [85].
- **Predictive Modeling of Pathogen Evolution:** Machine learning models capable of forecasting viral mutation trajectories (e.g., SARS-CoV-2, influenza) can enhance proactive public health interventions [86, 87].
- **Edge Computing for Field Deployments:** Developing lightweight bioinformatics pipelines that run on mobile devices or low-power hardware can improve genomic surveillance in remote and resource-limited settings [81, 88].

### Ethical and Regulatory Considerations

The use of genome sequencing technology including LRS in outbreak response raises critical ethical and regulatory concerns, including data privacy, biosecurity risks, and equitable access. Key recommendations include:

- **Ethical Guidelines for Pathogen Genomics: **Establishing global frameworks to regulate the ethical use of pathogen sequencing data, ensuring compliance with principles of equity and transparency [81].
- **Community Engagement in Genomic Surveillance:** Public health agencies must involve local communities in genomic surveillance efforts to foster trust and cooperation, particularly in outbreak-prone regions.
- **Regulatory Oversight of Pathogen Data Sharing:** Governments should implement policies that balance the need for rapid data sharing with concerns regarding national security and bioterrorism risks [81].

Privacy concerns are particularly salient in metagenomics, where sequencing samples from clinical or environmental sources can inadvertently capture host (human) genomic information. This raises significant ethical dilemmas around informed consent, re-identifiability, and data stewardship—especially when datasets are stored in open-access repositories. The blurred line between pathogen and host genomes challenges traditional notions of anonymity, requiring new models of ethical oversight and participant protection.

### Future Frontiers: Single-Molecule and Multi-Omics Integration

The next decade will likely witness the convergence of LRS with other omics technologies, expanding its applications beyond traditional genomic surveillance. Innovations include:

- **Single-Molecule Multi-Omics:** The integration of genomics, transcriptomics, epigenomics, and proteomics in a single sequencing run could provide a more holistic understanding of pathogen-host interactions [85].
- **Real-Time Epigenetic Surveillance:** ONT’s direct RNA sequencing capabilities enable real-time detection of viral epigenetic modifications, potentially revealing novel therapeutic targets [5, 89].
- **CRISPR-Based Pathogen Enrichment:** The combination of CRISPR technology with LRS can enhance targeted sequencing of outbreak-relevant genomic regions, improving diagnostic sensitivity [90].

## Conclusions

LRS technologies represent a transformative advancement in the field of infectious disease outbreak response. Their ability to generate contiguous, high-fidelity genomic sequences in real-time enables rapid identification of pathogens, characterization of antimicrobial resistance, and monitoring of viral evolution. The applications of LRS span various aspects of outbreak response, from genomic epidemiology to metagenomic studies, providing valuable insights into the dynamics of infectious diseases.

However, several limitations must be addressed to fully realize the potential of LRS in public health. These include error rates, computational challenges, standardization, cost considerations, and real-time application limitations. Ongoing research and technological advancements are essential for refining LRS methodologies and enhancing their accessibility and impact in outbreak response.

As the landscape of infectious disease genomics continues to evolve, LRS is a versatile tool for real-time pathogen surveillance, offering unprecedented opportunities to mitigate the impact of global health threats.

## Key References

- Raabe, N.J., et al., Real-time genomic epidemiologic investigation of a multispecies plasmid-associated hospital outbreak of NDM-5-producing Enterobacterales infections. Int J Infect Dis. 2024;142:106971.
  - This study presents a real-time genomic epidemiologic investigation of a multispecies plasmid-associated hospital outbreak of NDM-5-producing Enterobacterales infections. It highlights the importance of real-time sequencing in hospital outbreak management.
- Willett, B.J., et al., SARS-CoV-2 Omicron is an immune escape variant with an altered cell entry pathway. Nature Microbiology. 2022;7(8):1161–79.
  - Omicron is an immune escape variant of the SARS-CoV-2 virus with an altered cell entry pathway. The study shows how sequencing helps track viral evolution and inform public health interventions.
- Schwab, T.C., et al., Field evaluation of nanopore targeted next-generation sequencing to predict drug-resistant tuberculosis from native sputum in South Africa and Zambia. Journal of Clinical Microbiology. 2025;63(3):e01390-24.
  - This study implemented field evaluation of nanopore targeted next-generation sequencing to predict drug-resistant tuberculosis from native sputum in South Africa and Zambia. The authors demonstrate a real-world application of nanopore sequencing in TB control, with highly relevance for outbreak response.

## Data Availability

No datasets were generated or analysed during the current study.
